# Association between spectral electroencephalography power and autism risk and diagnosis in early development

**DOI:** 10.1002/aur.2518

**Published:** 2021-05-06

**Authors:** Scott Huberty, Virginia Carter Leno, Stefon J. R. van Noordt, Rachael Bedford, Andrew Pickles, James A. Desjardins, Sara Jane Webb, The BASIS Team, Mayada Elsabbagh

**Affiliations:** ^1^ Montreal Neurological Institute−Hospital, Azrieli Centre for Autism Research, McGill University Montréal Canada; ^2^ Institute of Psychiatry, Psychology & Neuroscience, King's College London London England UK; ^3^ Compute Ontario Toronto Ontario Canada; ^4^ Center on Child Health, Behavior and Development, Seattle Children's Research Institute Seattle Washington USA; ^5^ Centre for Brain and Cognitive Development England UK; ^6^ University of Bath Bath England UK

**Keywords:** autism spectrum disorders, EEG, infants, siblings

## Abstract

Autism spectrum disorder (ASD) has its origins in the atypical development of brain networks. Infants who are at high familial risk for, and later diagnosed with ASD, show atypical activity in multiple electroencephalography (EEG) oscillatory measures. However, infant‐sibling studies are often constrained by small sample sizes. We used the International Infant EEG Data Integration Platform, a multi‐site dataset with 432 participants, including 222 at high‐risk for ASD, from whom repeated measurements of EEG were collected between the ages of 3–36 months. We applied a latent growth curve model to test whether familial risk status predicts developmental trajectories of spectral power across the first 3 years of life, and whether these trajectories predict ASD outcome. Change in spectral EEG power in all frequency bands occurred during the first 3 years of life. Familial risk, but not a later diagnosis of ASD, was associated with reduced power at 3 months, and a steeper developmental change between 3 and 36 months in nearly all absolute power bands. ASD outcome was not associated with absolute power intercept or slope. No associations were found between risk or outcome and relative power. This study applied an analytic approach not used in previous prospective biomarker studies of ASD, which was modeled to reflect the temporal relationship between genetic susceptibility, brain development, and ASD diagnosis. Trajectories of spectral power appear to be predicted by familial risk; however, spectral power does not predict diagnostic outcome above and beyond familial risk status. Discrepancies between current results and previous studies are discussed.

**Lay Summary:**

Infants with an older sibling who is diagnosed with ASD are at increased risk of developing ASD themselves. This article tested whether EEG spectral power in the first year of life can predict whether these infants did or did not develop ASD.

## BACKGROUND

Recent evidence suggests that autism spectrum disorder (ASD) has its origins in the atypical development of brain networks (O'Reilly et al., 2017; Wang et al., 2013). Synaptic changes begin in the earliest stages of post‐natal life, and many of the genes implicated in ASD are also involved in the formation and regulation of synaptic pathways and neuronal connections (Parikshak et al., 2013). These findings have been supported by studies of infants at high familial risk for ASD (by virtue of having an older sibling with ASD), which report brain overgrowth and atypical development of white matter pathways in the first year of life in infants who are later diagnosed with ASD (Hazlett et al., 2017; Wolff et al., 2012). There is also evidence of early atypical spectral activity in ASD, as studies that use electroencephalography (EEG) to measure neural functioning have found reduced functional connectivity in gamma band oscillations, and greater functional connectivity in alpha band oscillations in infant‐siblings who later develop ASD (Orekhova et al., 2014; Righi et al., 2014). This early atypical connectivity may be due to differences in rates of axonal remodeling, leading to brain overgrowth and weakened long distance connections (O'Reilly et al., 2017).

EEG spectral power, a marker of cortical activity, has also been associated with ASD (Wang et al., 2013); however, its relation to ASD risk and development is not well understood. One longitudinal study of spectral power reported that infants at high‐risk (HR) of developing ASD (HR; infants who have an older sibling with ASD) exhibit lower EEG spectral power in several frequency bands at 6 months compared to those at low‐risk (LR; infants who have no siblings with an existing ASD diagnosis), and the two groups showed different trajectories over the first 2 years of life in several frequency bands (Tierney et al., 2012). A follow‐up study with the same sample found that the trajectories of absolute delta and gamma power differentiated those infants who would go on to develop ASD from those who did not (Gabard‐Durnam et al., 2019). Given that spectral power is associated with attention in infants and children (Orekhova et al., 2006), and is associated with language ability in the first years of life (Benasich et al., 2008; Levin et al., 2017, Wilkinson et al., 2019), understanding how spectral power relates to ASD risk and diagnosis will provide a better understanding of the developmental outcomes in this population.

A major challenge in the field is that most infant‐sibling studies have been limited by relatively small sample sizes. Given a recurrence rate of 20%, the majority of infant siblings do not go on to develop ASD (Constantino et al., 2010), meaning that, in most independent cohort studies, the subgroup of infants who develop ASD is relatively small. Furthermore, ASD is heterogenous; infants at HR who go on to a diagnosis differ substantially in their developmental outcomes across multiple domains (Charman et al., 2017).

These challenges to understanding the underlying brain mechanisms in ASD can be addressed with the use of multi‐study data‐platforms, which increase the sample size and capture greater heterogeneity between participants. With the development of the NIH's National Database for Autism Research (Payakachat et al., 2016), and specific to magnetic resonance imaging (MRI), the ABIDE database (Di Martino et al., 2014), data‐platforms are becoming increasingly utilized. Given the relatively small sample sizes of most infant EEG studies, particularly of infants at HR of ASD, the use of data‐platforms can advance our understanding of early development of brain networks in this population. In the current study, we examined early trajectories of EEG spectral power using the International Infant EEG Data Integration Platform (EEG‐IP; van Noordt et al., 2020). EEG‐IP includes 432 unique participants with multiple EEG recordings collected over the first 3 years of life, combining data sets from longitudinal infant sibling studies from three contributing sites (Boston Children's Hospital, in Boston, Massachusetts; University of London, in London, U.K; and the University of Washington, in Seattle, Washington). Each contributing site has published data from origin studies (Elsabbagh et al., 2012; Orekhova et al., 2014; Gabard‐Durnam et al., 2019; Levin et al., 2017; Righi et al., 2014; Tierney et al., 2012; Jones et al., 2015; Jones et al., 2016; Jones, Dawson, et al., 2017; Jones, Venema, et al., 2017), as well as contributed raw EEG and behavioral data to this repository. Upon entry into EEG‐IP, EEG recordings were processed into a standardized data‐state using the EEG‐IP Lossless Pipeline (Desjardins et al., 2020), and combined for analyses in this report.

The objective of this study was to examine the development of spectral power throughout the first 3 years of life as it relates to familial risk and diagnostic outcome. With the unprecedented sample size made available by EEG‐IP, we aimed to apply a statistical model that reflects the temporal relationship between genetic risk, trajectories of brain development, and ASD diagnosis. This analytical framework, using a latent growth curve model (GCM), assesses whether the trajectory of EEG spectral power can explain variation in diagnostic outcome beyond known familial risk status, and thus can expand upon previous studies that have found EEG spectral power to be a potential biomarker of ASD (Gabard‐Durnam et al., 2019; Wang et al., 2013).

Based on previous findings, we hypothesized that familial risk would predict the intercepts of absolute spectral power of all frequency bands, such that the intercepts would be lower in infants at high familial risk for ASD compared to infants with no familial risk for ASD (Tierney et al., 2012). We also hypothesized that familial risk status would predict the slopes of absolute power in all frequency bands, such that the slope would be steeper in infants at high familial risk for ASD compared to infants with no familial risk for ASD (Bosl et al., 2011; Tierney et al., 2012). In relation to ASD diagnosis, we hypothesized that the slope of absolute power in the lower frequency bands (Delta, Theta) would predict ASD outcome, such that the slope would be less steep in the HR infants that develop ASD, as compared to both HR infants that do not develop ASD, and LR infants that do not develop ASD (Gabard‐Durnam et al., 2019). Finally, while a study reported slightly higher absolute power in beta and gamma frequency bands in male infants as compared to female infants, a follow up study found that biological sex was not a significant predictor of spectral power (Gabard‐Durnam et al., 2019; Tierney et al., 2012). Given that no associations between absolute power and ASD risk or diagnosis have been found to be dependent on biological sex, we hypothesized that we would find biological sex differences in spectral power irrespective of ASD risk status, such that males would exhibit a higher intercept in the beta and gamma absolute power frequency bands than females, but a less steep slope (Tierney et al., 2012).

## METHODS AND MATERIALS

### Participants

Participants were infants who were either at HR for ASD by virtue of having an older diagnosed sibling, or LR controls with no family history of ASD. EEG‐IP includes 1382 EEG recordings from 432 unique infants from three contributing sites (Boston: 971; London: 188; Seattle: 223), spanning multiple age ranges (3–36 months). Each site conducted studies that were approved by their respective institutional review boards. The current analysis includes 1229 EEG recording sessions from 397 unique participants (208 male). Remaining infant recordings were excluded due to the following reasons: infant carrying familial risk for a condition other than ASD (risk for language disorder, *n* = 60), EEG recording missing the resting state paradigm (*n* = 38), recording falling outside the target age ranges set by the sites (*n* = 5). Fifty recordings were excluded for not containing a sufficient amount of resting data, which was determined to be a minimum of 32 s after data reduction, following previous guidelines (Gasser et al., 1985; Salinsky et al., 1991). Table 1 presents a summary of the number of participants.

**TABLE 1:** Available EEG recordings for current analysis, at each visit across sites; total of 1229 recordings on 397 unique participants. Numbers inside bracket in Boston row indicate EEG recordings using the EGI 65 channel Geodesic Net

Site	*n*	3 months	6 months	9 months	12 months	18 months	24 months	36 months	EEG recordings
Boston	219 (119 male)	50 (8)	141 (35)	159 (34)	162 (43)	121 (20)	122 (16)	128 (1)	883
Seattle	87 (54 male)		79		68	61			208
London	91 (35 male)		55		83				138
High Risk	214 (115 male)	34 (*20*)	136 (71)	84 (46)	165 (87)	105 (65)	70 (39)	73 (42)	
Low Risk	183 (93 male)	16 (10)	139 (71)	75 (41)	148 (70)	77 (44)	52 (25)	55 (26)	

*Note:* The italic values inside brackets in the High Risk and Low Risk rows indicate the number of EEG recordings from male participants.

Of the 397 participants, 214 infants (125 Boston; 48 London; 41 Seattle; 115 males total) were at HR for ASD. Among these, 61 infants (41 males) received a diagnosis of autism in toddlerhood. The remaining 183 infants (93 males) were in the LR group and had no family history of ASD. In the LR group, six infants (five males) received a diagnosis of autism in toddlerhood. To ascertain ASD diagnosis, the three sites used comparable measures, including the Autism Diagnostic Observation Schedule (ADOS; Lord et al., 2012; Lord et al., 2000), along with clinical judgment. For the London study, the ADOS‐2 was administered at both 24 and 36 months for the HR infants, and at 36 months for the LR infants. In the Seattle study, the ADOS was administered at 18 months and 24 months. In the Boston study, the ADOS was administered at 18 months, 24 months, and 36 months. Lastly, the three sites were comparable in the proportion of HR infants that went on to receive a diagnosis of ASD (Boston, 15%, London, 16%; Seattle: 13%).

### EEG data collection

All data in EEG‐IP were collected using Electrical Geodesics NetStation software. With the exception of initial recordings from Boston, which used the 64 channel Geodesic sensor net (159 recordings), all other recordings used the 128 channel Hydrocel net. See the section on power extraction below for details of standardization across nets.

In the Seattle and London samples, resting EEG (rs‐EEG) was collected while infants watched videos on a monitor while sitting on their caregiver's lap in a dark room. The Seattle videos consisted of a set of age appropriate brightly colored toys moving and producing sounds and a set with an adult woman facing the camera and singing nursery rhymes. The London sample consisted of these videos, and there was an additional third set of videos of age appropriate toys being activated by a human hand. The two video sets in the Seattle sample lasted 60 s each and the three video sets on the London sample lasted 30–40 s each. In the Boston sample, rs‐EEG was collected while infants sat on their caregiver's lap in a dimly lit room and an experimenter blew bubbles to sustain the child's attention. Rs‐EEG was collected for as long as the infants would sit calmly, an average of 3–4 min.

### Data standardization & reduction in EEG‐IP

In the EEG‐IP Platform, data were pre‐processed and standardized in order to be maximally compatible for cross‐site analysis. Open source solutions to technical constraints that typically impede successful integration were employed, including the Brain Imaging Data Structure extension to EEG (Gorgolewski et al., 2016; Pernet et al., 2019) and standardized pre‐processing using the EEG‐IP Lossless Pipeline (https://github.com/BUCANL/EEG-IP-L; Desjardins et al., 2020), which includes systematic pre‐processing procedures for identifying unreliable EEG signals and building comprehensive data annotation regarding signal quality.

The EEG‐IP Lossless Pipeline harmonizes data recordings by implementing data quality assessment procedures that are robust to project eccentricities in EEG data acquisition. The pipeline first addresses differences across datasets by executing *staging* scripts that are specific to each project, including procedures for the co‐registration of electrode coordinates to a common shared head surface, a robust average reference, and a 1 Hz high pass and notch filter (49–51 Hz in the London dataset, 59–61 Hz in the Boston and Seattle datasets). The staging scripts then flag extremely bad time periods and channels based on consistently outlying variance values. The rest of the pipeline quality assessments use confidence intervals of signal properties within each file to flag unusual time periods and channels. Each time that channels are flagged as problematic, the data are re‐referenced to interpolated channels on the shared co‐registered head surface. Following the scalp channel assessment, a robust Adaptive Mixture ICA procedure is performed.

After completion of the EEG‐IP Lossless pipeline, EEG recordings from each site that retained enough signal to be included in post‐processing were assessed for comparability (van Noordt et al., 2020). The proportion of time removed from data due to artifact was similar across sites, as was the distribution of data removed due to different properties (extreme voltage variance, low correlation with neighboring channels, artifact identified by ICA decomposition). The average channel retention (which ranged from 77–82%), and the spatial variance in both the retained and rejected independent components was also similar across datasets. Finally, a power spectrum profile of the EEG recordings showed that the EEG‐IP Lossless Pipeline resulted in similar profiles across datasets (van Noordt et al., 2020).

### Spectral power extraction

Measures of absolute and relative spectral power were computed from the standardized data state of EEG‐IP. Absolute power is the squared amplitude in a given frequency or frequency band. Relative power is the ratio of the absolute power in one frequency band compared to that of the total power spectrum across all frequencies of interest. Relative power thus reflects the relationship between frequency bands, as changes in one frequency band will affect others. While absolute power is ideal for studying activity in a specific frequency band independent of others, absolute power findings in ASD studies have been inconsistent compared to relative power findings (Wang et al., 2013).

Data were segmented into 4000 ms 50% overlapping epochs. EEG channels were interpolated to correspond to the 10–20 system—F7, Fpz, AF8, F3, Fz, F4, FT7, C3, Cz, C4, FT8, TP7, P3, Pz, P4, TP8, PO7, Oz, PO8. We were interested in the frontal (F3, Fz, F4) region, as a consistent finding in ASD literature is that the frontal region displays differences in spectral measures (Fox & Bell, 1990; Gabard‐Durnam et al., 2019; Levin et al., 2017; Mundy et al., 2000; Tierney et al., 2012).

**FIGURE 1:**
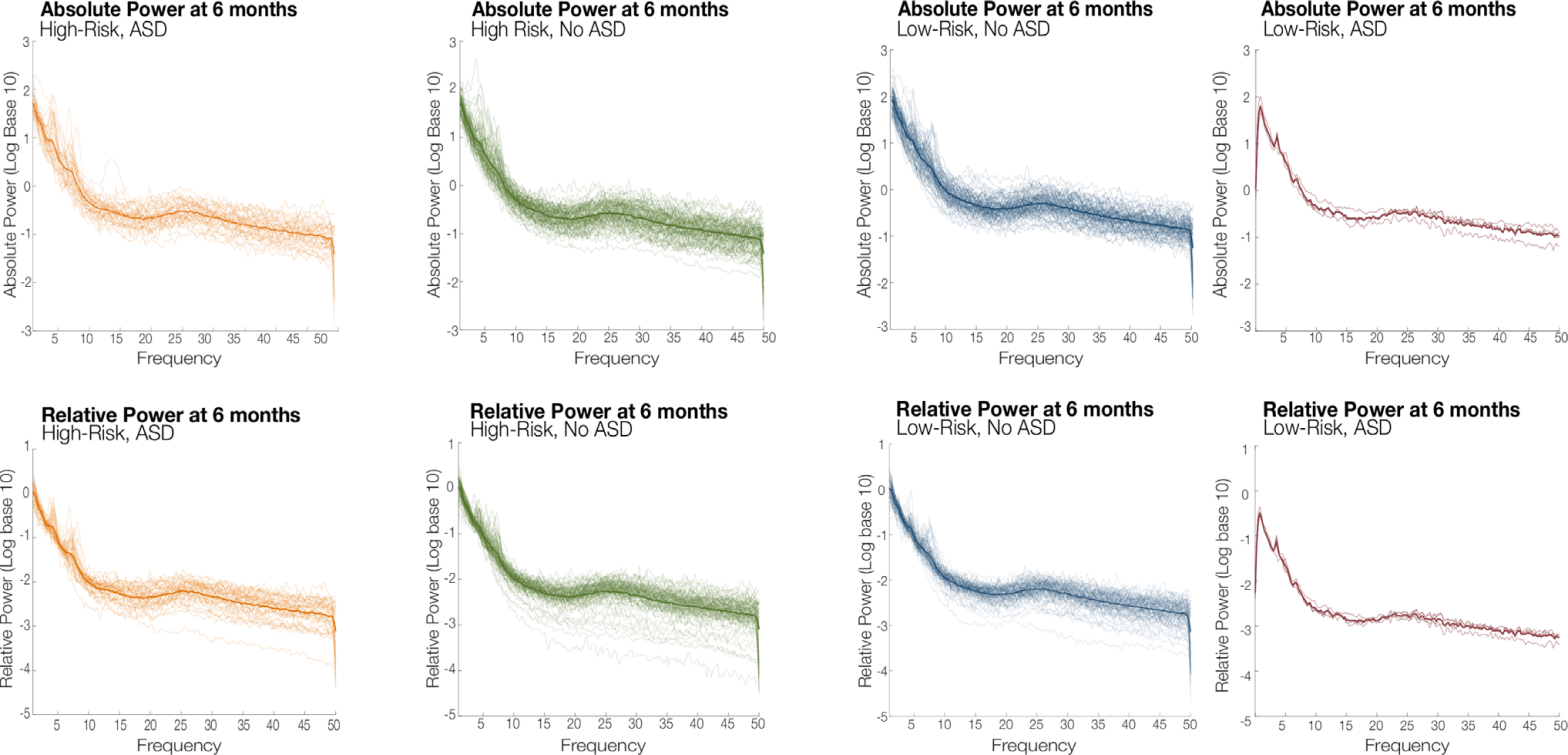
Power spectral densities at 6 months for each EEG recording by group for absolute and relative power

The frequency bands selected for analysis included delta (2–4 Hz), theta (4–6 Hz), low‐alpha (6–9 Hz), high‐alpha (9–13 Hz), beta (13–30 Hz), and gamma (30–50 Hz). For each electrode in the frontal region of interest (ROI), power spectral density of the area under the curve (trapezoidal numerical integration) was computed with the Welch method using the *pwelch* function in MATLAB. Having divided the signal into sections of equal length with 50% overlap, a Hamming window was applied before estimating a modified periodogram with a 0.25 Hz frequency bin resolution (4 s) for each segment. The 50% overlap of the segments is used to account for the fact that the Hamming window weighs the center of the data segment more heavily than the sidelobes, which are attenuated by 42.5 dB. Periodograms for all segments were then averaged to produce a final spectral estimate. Frequency band relative power was calculated by dividing the spectral power area under the curve (trapezoidal numerical integration) in a given band (e.g., delta = 2‐4 Hz) by the area under the curve of the total spectral range of interest (2–50 Hz). The relative power values of each channel were then averaged within the frontal ROI. Due to skewed distributions, absolute EEG values were log(10) transformed. See Figure 1 for group averaged and individual power spectral densities for all participants at the 6 month visit.

### Statistical analysis

The statistical analysis was conducted in Stata Version 14. A latent GCM was fit to the data using the gsem command, which allowed each occasion of measurement to have its own mean and error variance. Intercept (the initial level of EEG power at 3 months) and slope (the change in EEG power over time) were specified as latent variables that varied across individuals. Estimated individual trajectories were formed from systematic variation (e.g., the mean intercept and slope; fixed effects) combined with individual deviation (in intercept and linear slope) from the mean pattern of change (random effects). The mean slope was free, meaning that the fixed part of the model that describes the mean change was entirely unconstrained and allowed for different levels between sites. Thus, each individual's fitted trajectory is the addition of the systematic pattern appropriate to their site, and that individual's particular level and trend around that pattern. Estimated individual trajectories of spectral power are displayed in Figures S1 and S2. To aid convergence, EEG values were multiplied by 100.

**FIGURE 2:**
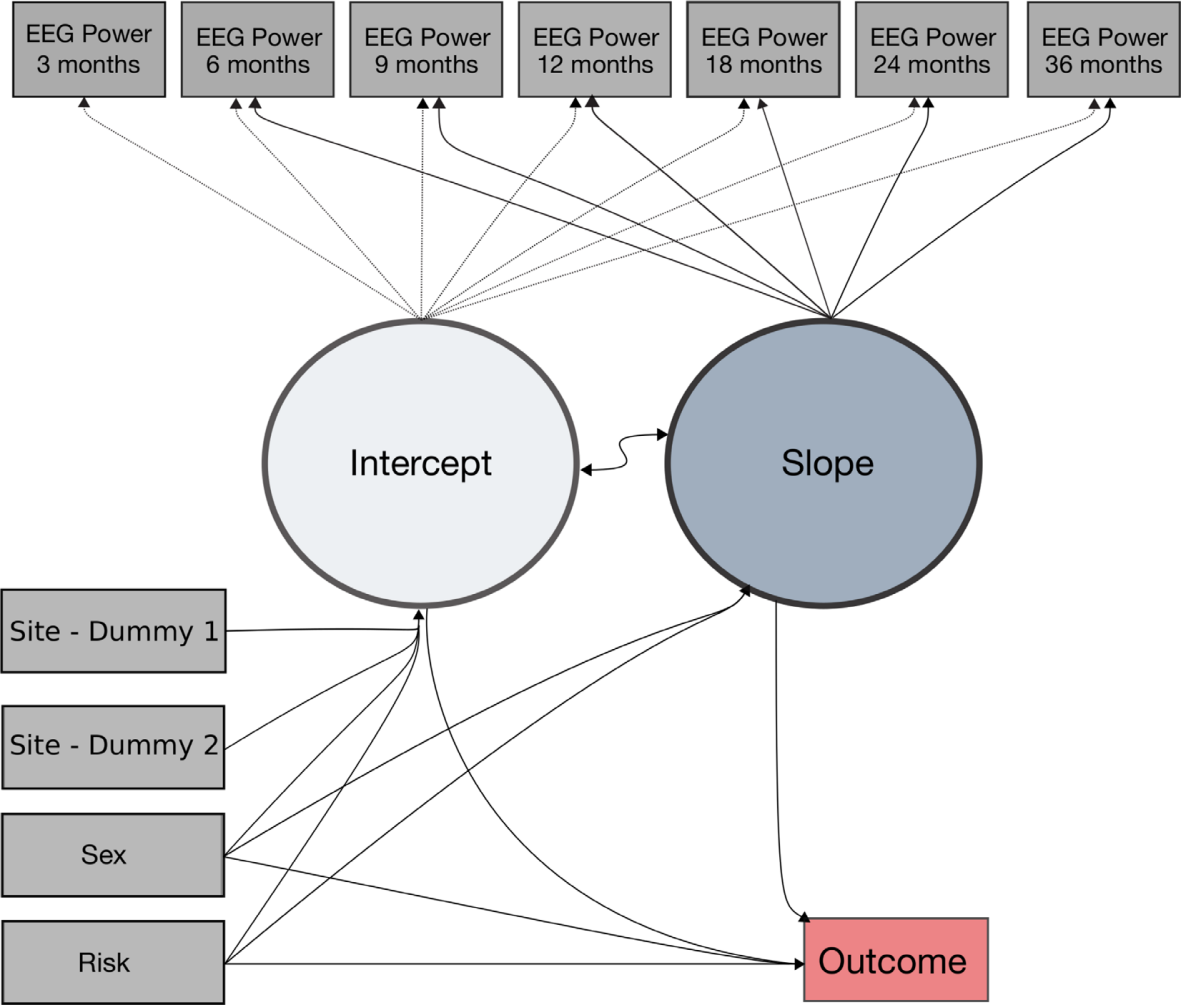
Growth curve model of fixed and random effect

Sex (male/female) and familial risk group (LR/HR) were specified as predictors of EEG intercept and slope, and in turn intercept and slope were specified as predictors of outcome (ASD diagnosis present/absent). Two dummy variables for site were also included as predictors of EEG intercept to account for between‐site differences in EEG collection, such as variations in recording parameters and paradigm. To ensure the robustness of findings, an additional model was run with site also included as a predictor of EEG slope; however, the results were unchanged, (Tables S1 and S2). Direct paths from sex and familial risk group to outcome were also specified (Figure 2), allowing for outcome differences not mediated by EEG power. As outcome was a binary variable (ASD versus no ASD), a binomial model with a logit link function was specified. Post‐estimation commands were used to test whether the variation over time in each power band was significantly different from zero (i.e., no change).

All models were estimated with full maximum likelihood to account for missing data under the “missing at random” assumption, under which missingness is assumed to relate only to observed variables in the model. Thus, for example, participants without a 3‐month visit have imputed intercept scores, which contributed less to the model as it knows that they are imputed rather than measured data. The inclusion of site accounts for the differing measurement schedules between sites, each with data missing by design. GCMs were fitted for each power band individually. Wald tests were used to assess the significance of paths. All reported path coefficients are unstandardized coefficients. The margins/marginsplot commands was used to plot marginal EEG profiles by familial risk and outcome group. Estimates of model fit were not available, as the gsem command does not provide these, similar to mixed‐effect models (comparable in approach to GCMs). In line with guidance on assessing the suitability of mixed‐effect models, we checked key assumptions were met (e.g., normality of residuals) and explored whether the specified model was a reasonable approximation of the raw data by inspecting correlations between observed and predicted values (Cheng et al., 2010). Correlations ranged from 0.75–0.83 for absolute power values and 0.61–0.77 for relative power values (averaged across all time points), suggesting the specified model was appropriate.

## RESULTS

**FIGURE 3:**
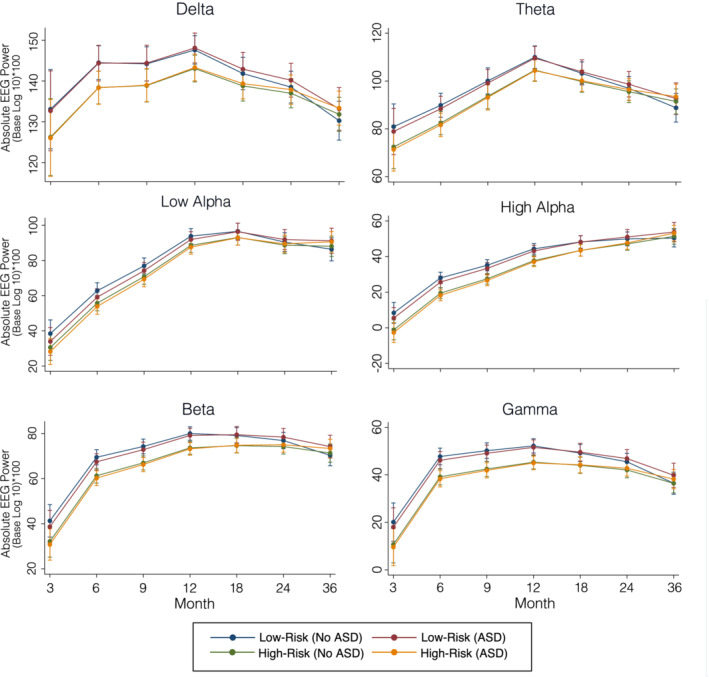
Growth curve models of absolute spectral power by group for each frequency band

**FIGURE 4:**
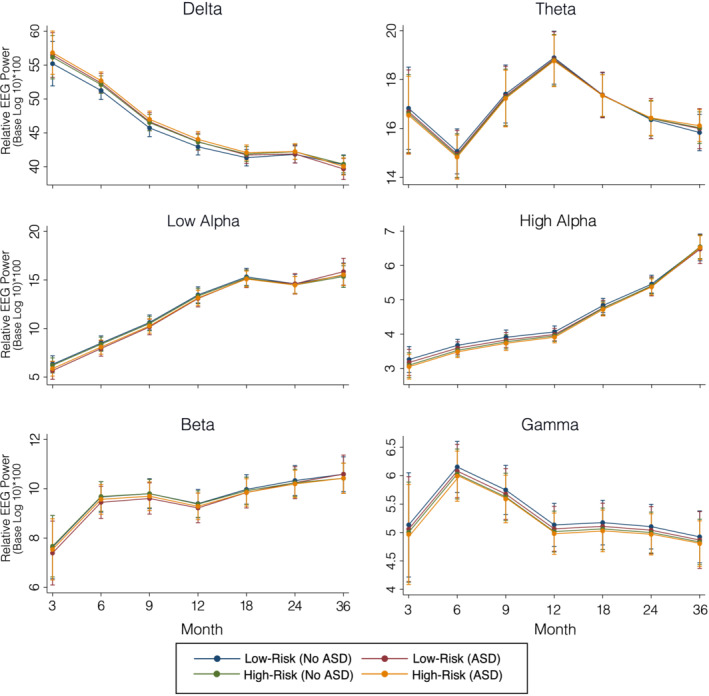
Growth curve models of relative spectral power for each frequency band

Trajectories of power in each frequency band for the four groups are shown in Figures 3 and 4: LR no ASD (LR‐no ASD), LR‐ASD, HR no ASD (HR‐no ASD), and HR ASD. Tables 2 and 3 present unstandardized parameter estimates and their associated significance levels. Overall, the change in EEG power over time was significantly different from zero in all frequency bands (all *p*s < 0.01), and from inspection of the graphs (Figures 3 and 4) it was clear that EEG power in some frequency bands was increasing linearly over time, most notable in the two alpha bands, while in others EEG power was decreasing over time, most notable in the delta band.

**TABLE 2:** Growth curve model (GCM) results for each absolute power band

	Absolute log transformed delta	Absolute log transformed theta	Absolute log transformed low‐alpha	Absolute log transformed high‐alpha	Absolute log transformed beta	Absolute log transformed gamma
Change over time	**χ** ^**2**^ **(6) = 67.86, *p* < 0.001**	**χ** ^**2**^ **(6) = 176.45, *p* < 0.001**	**χ** ^**2**^ **(6) = 515.03, *p* < 0.001**	**χ** ^**2**^ **(6) = 267.83, *p* < 0.001**	**χ** ^**2**^ **(6) = 200.78, *p* < 0.001**	**χ** ^**2**^ **(6) = 130.51, *p* < 0.001**
**Intercept on**						
Sex	*b* = −1.12, *p* = 0.640	*b* = −5.49, *p* = 0.064	*b* = **12.31, *p* < 0.001**	*b* = **−7.97, *p* < 0.001**	*b* = **−7.19, *p* < 0.001**	*b* = **−5.77, *p* = 0.006**
Risk	*b* = **−6.84, *p* = 0.004**	*b* = **−8.39, *p* = 0.005**	*b* = **−7.73, *p* = 0.004**	*b* = **−9.42, *p* < 0.001**	*b* = **−9.03, *p* < 0.001**	*b* = **−9.36, *p* < 0.001**
Site—Dummy 1 (London)	*b* = **−5.03, *p* = 0.044**	*b* = 6.15, *p* = 0.067	*b* = **6.34, *p* = 0.036**	*b* = **−4.84, *p* = 0.021**	*b* = 1.97, *p* = 0.359	*b* = 1.78, *p* = 0.413
Site—Dummy 2 (Seattle)	*b* = **11.70, *p* < 0.001**	*b* = **−12.45, *p* < 0.001**	*b* = **−7.21, *p* = 0.012**	*b* = **−12.70, *p* < 0.001**	*b* = **−5.90, *p* = 0.004**	*b* = **−8.51, *p* < 0.001**
**Slope on**						
Sex	*b* = **3.30, *p* = 0.023**	*b* = **5.84, *p* = 0.001**	*b* = **9.33, *p* < 0.001**	*b* = **6.27, *p* < 0.001**	*b* = **6.50, *p* < 0.001**	*b* = **5.48, *p* < 0.001**
Risk	*b* = **3.02, *p* = 0.041**	*b* = **3.97, *p* = 0.026**	*b* = 3.39, *p* = 0.067	*b* = **3.75, *p* = 0.010**	*b* = **3.61, *p* = 0.011**	*b* = **3.36, *p* = 0.024**
**Outcome on**						
Sex	*b* = **1.19, *p* = 0.014**	*b* = **1.15, *p* = 0.017**	*b* = **1.44, *p* = 0.007**	*b* = **1.04, *p* = 0.021**	*b* = **1.00, *p* = 0.032**	*b* = 0.88, *p* = 0.055
Risk	***b* = 2.68, *p* < 0.001**	***b* = 2.62, *p* < 0.001**	***b* = 2.71, *p* < 0.001**	***b* = 2.52, *p* < 0.001**	***b* = 2.53, *p* < 0.001**	***b* = 2.49, *p* < 0.001**
Intercept	*b* = −0.02, *p* = 0.247	*b* = −0.01, *p* = 0.294	*b* = 0.01, *p* = 0.505	*b* = −0.01, *p* = 0.540	*b* = 0.01, *p* = 0.834	*b* = 0.01, *p* = 0.586
Slope	*b* = −0.09, *p* = 0.345	*b* = −0.05, *p*=0.371	*b* = −0.04, *p* = 0.200	*b* = −0.03, *p* = 0.523	*b* = −0.02, *p* = 0.799	*b* = 0.01, *p* = 0.874

*Note:* The values marked in bold are statistically significant.

**TABLE 3:** Growth curve model (GCM) results for each relative power band

	Relative delta	Relative theta	Relative low alpha	Relative high alpha	Relative beta	Relative gamma
Change over time	**χ** ^**2**^ **(6) = 287.33, *p* < 0.001**	**χ** ^**2**^ **(6) = 88.68, *p* < 0.001**	**χ** ^**2**^ **(6) = 351.59, *p* < 0.001**	**χ** ^**2**^ **(6) = 158.09, *p* < 0.001**	**χ** ^**2**^ **(6) = 18.27, *p* = 0.006**	**χ** ^**2**^ **(6) = 32.99, *p* < 0.001**
**Intercept on**						
Sex	*b* = **3.52, *p* < 0.001**	*b* = −0.38, *p* = 0.497	*b* = **−1.78, *p* < 0.001**	*b* = **−0.24, *p* = 0.025**	*b* = −0.69, *p* = 0.052	*b* = −0.21, *p* = 0.401
Risk	*b* = 0.91, *p* = 0.229	*b* = −0.21, *p* = 0.710	*b* = −0.09, *p* = 0.827	*b* = −0.16, *p* = 0.128	*b* = 0.04, *p* = 0.905	*b* = −0.13, *p* = 0.602
Site—Dummy 1 (London)	*b* = **−5.76, *p* < 0.001**	*b* = **2.42, *p* < 0.001**	*b* = **1.82, *p* = 0.001**	*b* = **−0.44, *p* < 0.001**	*b* = 0.24, *p* = 0.573	*b* = 0.26, *p* = 0.340
Site—Dummy 2 (Seattle)	*b* = **−1.88, *p* = 0.016**	*b* = −70, *p* = 0.221	*b* = 0.80, *p* = 0.103	*b* = **−0.24, *p* = 0.027**	*b* = **1.03, *p* = 0.008**	*b* = 0.29, *p* = 0.241
**Slope on**						
Sex	*b* = **−1.92, *p* < 0.001**	*b* = 0.30, *p* = 0.249	*b* = **1.01, *p* = 0.002**	*b* = 0.04, *p* = 0.711	*b* = 0.25, *p* = 0.188	*b* = 0.02, *p* = 0.893
Risk	*b* = −0.30, *p* = 0.487	*b* = 0.14, *p* = 0.574	*b* = −0.02, *p* = 0.949	*b* = 0.07, *p* = 0.531	*b* = −0.08, *p* = 0.694	*b* = 0.01, *p* = 0.922
**Outcome on**						
Sex	*b* = 0.78, *p* = 0.134	*b* = 0**.85, *p* = 0.008**	*b* = 1.29, *p* = 0.052	*b* = 0**.85, *p* = 0.007**	*b* = 0**.87, *p* = 0.006**	*b* = 0**.98, *p* = 0.018**
Risk	*b* = **2.45, *p* < 0.001**	*b* = **2.47, *p* < 0.001**	*b* = **2.49, *p* < 0.001**	*b* = **2.42, *p* < 0.001**	*b* = **2.45, *p* < 0.001**	*b* = **2.64, *p* < 0.001**
Intercept	*b* = −0.04, *p* = 0.317	*b* = −0.07, *p* = 0.297	*b* = 0.13, *p* = 0.301	*b* = −0.12, *p* = 0.747	*b* = 0.10, *p* = 0.153	*b* = 0.20, *p* = 0.336
Slope	*b* = −0.12, *p* = 0.558	*b* = 0.10, *p* = 0.734	*b* = −0.11, *p* = 0.691	*b* = 0.35, *p* = 0.406	*b* = 0.40, *p* = 0.145	*b* = 1.95, *p* = 0.355

*Note:* The values marked in bold are statistically significant.

### Absolute power

ASD risk and outcome: There was a main effect of familial risk on intercept across all frequency bands (Table 2; Figure 3), such that the HR group showed lower power than the LR group (all *p*s < 0.01). There was also a main effect of familial risk on the slopes of delta, theta, high‐alpha, beta, and gamma, such that the HR group showed a steeper increase in power between 3 months and 36 months (all *p*s < 0.05). There were no effects of absolute power intercept or slope on ASD outcome. To demonstrate that our findings of risk on intercept were not influenced by study site, we re‐ran the model using only the 6–36 month visit data so that intercept was defined at 6 months (a visit that included data from all study sites; Tables S3 and S4), and the pattern of results remained unchanged.

Sex: There was a main effect of sex on the intercepts and slopes of high‐alpha, beta, and gamma such that females showed a lower intercept (i.e. lower power) and a steeper slope between 3 and 36 months as compared to males (see Table 2; all *p*s < 0.05). There was also a main effect of sex on the intercept and slope of low‐alpha, such that for low‐alpha, females showed a higher intercept (i.e. higher power) and a less steep slope between 3 months and 36 months.

### Relative power

ASD risk and outcome: There were no effects of risk on either the intercept or slope of relative power (all *p*s >0.10; see Table 3 and Figure 4). There were no effects of relative power intercept or slope on ASD outcome (all *p*s > 0.05; see Table 3).

**FIGURE 5:**
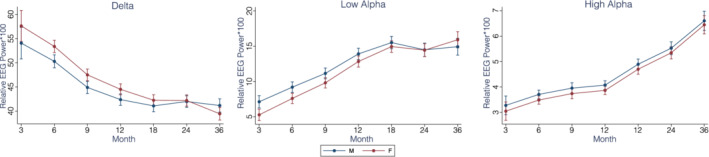
Growth curve models of relative spectral power by biological sex for the delta and alpha bands

Sex: There was a main effect of sex on delta, low‐alpha, and high‐alpha intercept such that at 3 months, females had a higher delta power, but lower low‐alpha and high‐alpha power (all *p*s <0.05; see Figure 5). There was also a significant association between sex and slope in the delta and low‐alpha frequencies, indicating that males and females had different trajectories of change over time in these power bands.

## DISCUSSION

Accumulating evidence suggests that subtle neural markers of ASD risk in the first year of life are present prior to the emergence of overt behavioral symptoms in childhood (Elsabbagh, 2020; Jones et al., 2014; Wolff et al., 2018). A more complex picture is now emerging that reveals the challenges of modeling trajectories between infants at risk for ASD who do and do not go on to receive a diagnosis, and how these compare to LR infants. Infants at risk who do not develop ASD present an overlapping set of neural and behavioral risk signs with those that do develop ASD (Elsabbagh & Johnson, 2016; Jones et al., 2014; Szatmari et al., 2016). Preliminary evidence suggests that protective influences in some infants may lead to neural re‐organization during early development and mitigates the impact of emerging symptoms on the developing brain (Elsabbagh, 2020).

In the current study, we investigated developmental changes in spectral power over the first 3 years of life using an integrated data‐platform comprising of three independent datasets with infants at‐risk of developing ASD and typically developing infants. We found that HR infants exhibited lower absolute, but not relative EEG power in all frequency bands as compared to LR infants at 3 months, with converging trajectories across the first 3 years of life. In contrast, we did not find evidence of significant differences in the intercept or slope of EEG spectral power between infants who developed ASD and those who did not.

We also documented sex differences in developmental EEG trajectories; despite autism being more prevalent in boys, these differences extended to both risk groups (although we note we did not test for a diagnosis‐by‐sex interaction). Females exhibited lower values of absolute power in the high‐alpha, beta, and gamma bands, and steeper slopes in these bands. There was also a main effect of sex on the relative delta, low‐alpha, and high‐alpha power intercepts such that at 3 months, females had a higher delta power, but lower low‐alpha and high‐alpha power (all *p*s <0.05; see Figure 5). There was also a significant association between sex and slope in the relative delta and low‐alpha frequencies, indicating that males and females had different trajectories of change over time in these power bands. Here too, sex differences converge where females and males become indistinguishable over the course of the first years of life. Considering that there is a lower prevalence of ASD in females, and that sex differences appear during early development in both typically and atypically developing infants across a range of domains (Messinger et al., 2015), these findings may signal underlying processes that promote better than expected outcomes in terms of ASD diagnosis in female infants at risk for ASD (Bedford et al., 2016; Elsabbagh, 2020). Additional research into the development of female infants at risk for ASD is needed.

Our findings of lower global absolute power at 3 months in the HR infants, which converges with the LR infants over the first years of life, is consistent with previous findings in infants at high familial risk for ASD (Gabard‐Durnam et al., 2019; Tierney et al., 2012). We also extended previous findings to examine relative power and did not find evidence to suggest that the proportion of total spectral power contributed by individual frequency bands is affected by familial risk. As such, differences in absolute power could in part be due to abnormal trajectories of axonal pruning and white matter maturation reported in ASD populations (O'Reilly et al., 2017; Wolff et al., 2012), possibly resulting in less neuronal synchrony and thus globally lower spectral power.

Yet, instead of differentiating the at‐risk infants who go on to develop ASD, risk trajectories of spectral power on the whole appear to converge with LR controls by 36 months. In the delta and theta frequency bands, the HR‐ASD group exhibited lower absolute power than the LR groups at 3 months, but by 36 months the groups converge. Follow‐up studies into later stages of development are needed to examine whether the groups would bifurcate again later in development, as suggested in studies with autistic adults (Wang et al., 2013).

Alternatively, our findings may suggest that achieving the potential utility of spectral power as an indicator of underlying brain mechanisms in ASD will rely on a shift away from categorical risk and diagnostic outcomes toward a more dimensional approach. Given the substantial variation among individuals with ASD in developmental outcomes, cognitive and behavioral domains may be more closely associated with atypical early brain development than a binary diagnostic ASD outcome. Additional genetic information could help to further stratify infant‐sibling samples, leading to a better understanding of the developmental process by which genetic susceptibility leads to atypical brain development and altered outcomes. Previous studies suggest that simplex ASD (in which only one sibling develops ASD) is associated with rare de novo mutations, while multiplex ASD (in which multiple siblings develop ASD) is more often associated with inherited genetic variants (D'Abate et al., 2019; McDonald et al., 2019), and higher risk for challenges in cognitive ability in childhood (McDonald et al., 2019). Including this information in future studies could aid in their accuracy in predicting outcomes in infants at risk for ASD.

Our null finding with regard to diagnostic outcomes show some discrepancies with previous findings that identified early emerging differences in EEG measures. A previous systematic review of a wider range of EEG measures used across the lifespan in autism found only few consistencies across a very large number of studies (O'Reilly et al., 2017), potentially due to sample heterogeneity, variation in pre and post‐processing pipelines, and variation in statistical models. While some of these challenges, such as sample heterogeneity, can be addressed with the use of data‐platforms, further work in our field is needed to standardize EEG collection protocols and processing pipelines, and so this study was limited in its ability to perform direct comparisons to previous studies.

With respect to sample heterogeneity, we pooled data across three sites (van Noordt et al., 2020), and similar discrepancies have been reported in previous studies utilizing pooled datasets (Bigdely‐Shamlo et al., 2020; Traut et al., 2018), possibly due to the fact that brain measures are particularly sensitive to factors such as age, sex, and IQ, which may give rise to significant findings in smaller samples (Traut et al., 2018). Further, despite the progress made in standardizing pooled datasets, EEG acquisition protocols across sites cannot be controlled retroactively. While the Boston dataset collected rs‐EEG while an experimenter blew bubbles, the other studies played videos. Each of these protocols introduces varying amounts of (social) engagement, which may impact spectral power profiles (Jones et al., 2015). However, we included site as a covariate in our model to take into account site differences in EEG parameters and collection schedules. As a secondary analysis, we tested for differences in the effect of risk between sites (Table S5). All risk‐by‐site interactions were nonsignificant, suggesting that our finding of risk predicting absolute power intercept and slope was not driven by any individual site. Nevertheless, the field would benefit from further standardization in data collection protocols and targeted comparisons of various EEG acquisition methods that can impact the results (Noreika et al., 2020; Webb et al., 2015).

In regard to pre‐processing EEG data, our approach of developing a standardized pre‐processing pipeline that was applied across the EEG‐IP datasets (Desjardins et al., 2020; https://github.com/BUCANL/EEG-IP-L) is part of the more general progress being made in standardizing EEG acquisition and analysis (Islam et al., 2016; Pernet et al., 2019; Webb et al., 2015). Other pipelines that are suitable for developmental populations where acquisition is brief and contains a higher proportion of artifact are also available (Debnath et al., 2020; Gabard‐Durnam et al., 2018; Leach et al., 2020; Levin et al., 2018). While these developments are a significant improvement over conventional approaches that predominantly rely on manual inspection for artifact removal, it remains unclear how choice among a multitude of processing pipelines may affect downstream EEG analyses (Robbins et al., 2020).

Finally, in relation to statistical modeling, our study adopted a theoretically driven approach aiming to understand the developmental mechanisms by which genetic risk may lead to atypical brain development, and in turn, predict ASD outcome. We applied a latent GCM, as it allowed us to specify whether genetic susceptibility predicts trajectories of spectral power development, and whether these trajectories predict ASD outcome, thus reflecting the temporal relationship between risk status, brain development, and ASD diagnosis. In this model, spectral power is specified to mediate the association between risk and ASD outcome, thus it must be a more proximal marker of outcome than familial risk alone to be considered statistically significant. In contrast, similar ASD studies have applied data‐driven approaches to differentiate participants by diagnostic outcomes (Bosl et al., 2018; Dickinson et al., 2018; Gabard‐Durnam et al., 2019; Jamal et al., 2014). Data‐driven models are important for the identification of potential biomarkers; however, they are often agnostic to the developmental nature of the condition, testing whether a parameter or set of parameters can predict risk status and outcome independently. These two approaches may show different results because they ask distinct (but complementary) questions (Botvinik‐Nezer et al., 2020; Ewen et al., 2019; McDermott et al., 2013).

Taken together, The current study investigated developmental changes in spectral power using an integrated data‐platform comprising of the largest sample to date of EEG recordings from at‐risk infants over the first 3 years of life. Results suggest that although developmental changes in absolute frontal EEG power may be one manifestation of familial risk, spectral power was not predictive of diagnostic outcome. Future research should take into consideration how differences in sample ascertainment, EEG assessment, and statistical approach may contribute to heterogenous patterns of results.

## DISCLOSURE OF INTERESTS

No competing interests were disclosed.

## ETHICS STATEMENT

Datasets used in EEG‐IP were originally approved by each institution's respective Ethics Review Board. De‐identified data were submitted to EEG‐IP via data‐transfer agreements between McGill University and each dataset's institution of origin. Authors have no financial conflicts of interest to declare.

## Supporting information

**AppendixS1**: Supporting information
