# Supplementary material for: Association between spectral electroencephalography power and autism risk and diagnosis in early development
Source: Autism Res. 2021 May 6;14(7):1390–403. doi: 10.1002/aur.2518 (PMC8360065; doi:10.1002/aur.2518)
Supplement: Supplementary file 1 — AppendixS1: Supporting information [file AUR-14-1390-s001.docx]

**Supplementary Materials**

Supplementary Materials Table 1: Growth curve model (GCM) results for each absolute power band, specifying study site as a predictor of slope

|  | **Absolute log transformed delta** | **Absolute log transformed theta** | **Absolute log transformed low-alpha** | **Absolute log transformed high-alpha** | **Absolute log transformed beta** | **Absolute log transformed gamma** |
| --- | --- | --- | --- | --- | --- | --- |
| **Intercept on** | | | | | | |
| Sex | b=-1.24, p=.607 | b=-5.50, p=.064 | **b=-12.32, p<.001** | **b=-7.98, p<.001** | **b=-7.12, p=.001** | **b=-5.64, p=.007** |
| Risk | **b=-6.77, p=.005** | **b=-8.39, p=.005** | **b=-7.73, p=.004** | **b=-9.35, p<.001** | **b=-9.03, p<.001** | **b=-9.34, p<.001** |
| Site – Dummy 1 (London) | b=-4.94, p=.347 | b=5.23, p=.329 | b=6.20, p=.170 | b=.52, p=.896 | b=7.49, p=.089 | **b=15.53, p=.001** |
| Site – Dummy 2 (Seattle) | b=-7.45, p=.076 | **b=-12.84, p=.005** | b=-6.95, p=.080 | **b=-9.18, p=.002** | b=-4.46, p=.173 | b=-5.42, p=.124 |
| **Slope on** | | | | | | |
| Sex | **b=3.36, p=.021** | **b=5.84, p=.001** | **b=9.34, p<.001** | **b=6.25, p<.001** | **b=6.43, p<.001** | **b=5.35, p<.001** |
| Risk | **b=2.97, p=.045** | **b=3.96, p=.026** | b=3.39, p=.068 | **b=3.69, p=.011** | **b=3.61, p=.011** | **b=3.36, p=.024** |
| Site – Dummy 1 (London) | b=.16, p=.982 | b=1.53, p=.830 | b=.29, p=.960 | b=-8.86, p=.122 | b=-9.00, p=.154 | **b=-22.25, p=.002** |
| Site – Dummy 2 (Seattle) | b=-4.87, p=.320 | b=.48, p=.909 | b=-.35, p=.925 | b=-4.97, p=.117 | b=-1.89, p=.587 | b=-3.98, p=.309 |
| **Outcome on** | | | | | | |
| Sex | **b=.97, p=.007** | **b=1.13, p=.026** | **b=1.44, p=.007** | **b=.89, p=.017** | **b=.91, p=.017** | **b=.88, p=.007** |
| Risk | **b=2.49, p<.001** | **b=2.61, p<.001** | **b=2.71, p<.001** | **b=2.43, p<.001** | **b=2.48, p<.001** | **b=2.50, p<.001** |
| Intercept | b=-.01, p=.341 | b=-.01, p=.321 | b=.01, p=.504 | b=-.01, p=.579 | b=.01, p=.772 | b=.01, p=.588 |
| Slope | b=-.03, p=.635 | b=-.05, =.431 | b=-.05, p=.203 | b=-.01, p=.765 | b=.01, p=.991 | b=.01, p=.668 |

Supplementary Materials Table 2: Growth curve model (GCM) results for each relative power band, specifying study site as a predictor of slope

|  | **Relative delta** | **Relative theta** | **Relative low alpha** | **Relative high alpha** | **Relative beta** | **Relative gamma** |
| --- | --- | --- | --- | --- | --- | --- |
| **Intercept on** | | | | | | |
| Sex | **b=3.45, p<.001** | b=-.39, p=.489 | **b=-1.77, p<.001** | **b=-.24, p=.030** | b=-.35, p=.326 | b=-.17, p=.481 |
| Risk | b=.95, p=.208 | b=-.20, p=.713 | b=-.10, p=.813 | b=-.17, p=.122 | b=.32, .377 | b=-.14, p=.551 |
| Site – Dummy 1 (London) | **b=-6.46, p<.001** | b=1.12, p=.351 | b=1.24, p=.069 | b=-.08, p=.712 | b=.65, p=.359 | **b=1.44, p=.028** |
| Site – Dummy 2 (Seattle) | b=.42, p=.744 | b=-1.11, p=.192 | b=.24, p=.723 | b=-.16, p=.326 | b=.62, p=.376 | b=-.29, p=.514 |
| **Slope on** | | | | | | |
| Sex | **b=-1.87, p<.001** | b=.30, p=.243 | **b=1.00, p=.002** | b=.03, p=.773 | b=.34, p=.084 | b=.01, p=.995 |
| Risk | b=-.32, p=.459 | b=.15, p=.573 | b=-.01, p=.968 | b=.07, p=.520 | b=.07, p=.735 | b=.02, p=.862 |
| Site – Dummy 1 (London) | b=1.55, p=.537 | b=2.94, p=.193 | b=1.18, p=.261 | b=-.66, p=.056 | b=-.12, p=.895 | **b=-1.98, p=.038** |
| Site – Dummy 2 (Seattle) | **b=-2.80, p=.026** | b=.56, p=.525 | b=.96, p=.212 | b=-.13, p=.470 | b=.98, p=.187 | b=.71, p=.117 |
| **Outcome on** | | | | | | |
| Sex | **b=.87, p=.024** | **b=.87, p=.005** | **b=1.54, p=.036** | **b=.83, p=.010** | **b=.88, p=.007** | **b=.92, p=.004** |
| Risk | **b=2.48, p<.001** | **b=2.48, p<.001** | **b=2.52, p<.001** | **b=2.41, p<.001** | **b=2.64, p<.001** | **b=2.51, p<.001** |
| Intercept | b=-.04, p=.292 | b=-.09, p=.172 | b=.21, p=.3226 | b=-.15, p=.712 | b=.10, p=.197 | b=.22, p=.134 |
| Slope | b=-.07, p=.489 | b=.03, p=.815 | b=-.21, p=.466 | b=.26, p=.470 | b=.32, p=.286 | b=.20, p=.306 |

Supplementary Materials Table 3: Growth curve model (GCM) results for each absolute power band for visits between 6 and 36 months; intercept: 6 months, slope: 6-36 months.

|  | **Absolute log transformed delta** | **Absolute log transformed theta** | **Absolute log transformed low-alpha** | **Absolute log transformed high-alpha** | **Absolute log transformed beta** | **Absolute log transformed gamma** |
| --- | --- | --- | --- | --- | --- | --- |
| Change over time | **χ²(5)=56.15, p<.001** | **χ²(5)=1116.71, p<.001** | **χ²(5)=295.03, p<.001** | **χ²(5)=152.66, p<.001** | **χ²(5)=89.73, p<.001** | **χ²(5)=72.80, p<.001** |
| Intercept on | | | | | | |
| Sex | b=-.13, p=.952 | b=-3.20, p=.252 | **b=-9.87, p<.001** | **b=-6.48, p<.001** | **b=-5.69, p=.002** | **b=-4.59, p=.014** |
| Risk | **b=-6.23, p=.004** | **b=-7.66, p=.006** | **b=-7.05, p=.005** | **b=-8.38, p<.001** | **b=-8.12, p<.001** | **b=-8.53, p<.001** |
| Site – Dummy 1 (London) | b=-4.78, p=.232 | b=5.07, p=.275 | b=6.29, p=.099 | b=-1.93, p=.494 | b=5.02, p=.110 | **b=9.83, p=.003** |
| Site – Dummy 2 (Seattle) | **b=-8.98, p=.008** | **b=-12.82, p=.001** | **b=-7.25, p=.038** | **b=-10.66, p=.000** | b=-5.07, p=.055 | **b=-6.47, p=.020** |
| Slope on | | | | | | |
| Sex | **b=3.17, p=.032** | **b=5.29, p=.003** | **b=9.35, p<.001** | **b=6.37, p<.001** | **b=6.65, p=.000** | **b=5.64, p<.001** |
| Risk | **b=3.09, p=.039** | **b=4.10, p=.026** | b=3.41, p=.074 | **b=3.59, p=.017** | **b=3.51, p=.015** | **b=3.30, p=.029** |
| Site – Dummy 1 (London) | b=-.06, p=.993 | b=3.10, p=.753 | b=-.31, p=.963 | b=-8.84, p=.117 | b=-8.80, p=.167 | **b=-22.13, p=.002** |
| Site – Dummy 2 (Seattle) | b=-5.27, p=.242 | b=.29, p=.951 | b=-.26, p=.949 | b=-4.91, p=.121 | b=-1.88, p=.591 | b=-3.99, p=.308 |
| Outcome on | | | | | | |
| Sex | **b=.93, p=.005** | **b=.96, p=.017** | **b=1.26, p=.008** | **b=.86, p=.020** | **b=.93, p=.017** | **b=.89, p=.007** |
| Risk | **b=2.44, p<.001** | **b=2.49, p<.001** | **b=2.61,p<.001** | **b=2.40, p p<.001** | **b=2.49, p<.001** | **b=2.51, p<.001** |
| Intercept | b=-.01, p=.284 | b=-.01, p=.237 | b=.01, p=.713 | b=-.01, p=.494 | b=.01, p=.746 | b=.01, p=.518 |
| Slope | b=-.01, p=.784 | b=-.02, p=.663 | b=-.03, p=.296 | b=-.01, p=.910 | b=-.01, p=.947 | b=.01, p=.708 |

Supplementary Materials Table 4: Growth curve model (GCM) results for each relative power band for visits between 6 and 36 months; intercept: 6 months, slope: 6-36 months.

|  | **Relative delta** | **Relative theta** | **Relative low-alpha** | **Relative high-alpha** | **Relative beta** | **Relative gamma** |
| --- | --- | --- | --- | --- | --- | --- |
| Change over time | χ²(5)=149.92, p<.001 | χ²(5)=89.92, p<.001 | χ²(5)=174.94, p<.001 | χ²(5)=146.12, p<.001 | χ²(5)=8.32, p=.139 | χ²(5)=21.61, p<.001 |
| Intercept on | | | | | | |
| Sex | **b=2.98, p<.001** | b=-.07, p=.892 | **b=-1.90, p<.001** | **b=-.25, p=.012** | b=-.65, p=.058 | b=-.23, p=.313 |
| Risk | b=.84, p=.232 | b=-.16, p=.761 | b=-.05, p=.914 | b=-.14, p=.144 | b=.07, p=.827 | b=-.12, p=.606 |
| Site – Dummy 1 (London) | **b=-6.03, p<.001** | **b=1.85, p=.025** | **b=1.52, p=.018** | b=-.24, p=.114 | b=.41, p=.476 | **b=.93, p=.038** |
| Site – Dummy 2 (Seattle) | b=-.23, p=.824 | b=-1.04, p=.148 | b=.48, p=.427 | b=-.19, p=.159 | b=.63, p=.217 | b=-.11, p=.764 |
| Slope on | | | | | | |
| Sex | **b=-1.89, p=.000** | b=.18, p=.506 | b=1.35, p=.000 | b=.05, p=.623 | b=.27, p=.156 | b=.05, p=.725 |
| Risk | b=-.29, p=.506 | b=.15, p=.587 | b=-.07, p=.837 | b=.06, p=.59 | b=-.12, p=.546 | b=.01, p=.972 |
| Site – Dummy 1 (London) | b=1.54. p=.551 | b=2.94, p=.187 | b=.98, p=.434 | b=-.68, p=.045 | b=-.59, p=.596 | **b=-1.98, p=.037** |
| Site – Dummy 2 (Seattle) | **b=-2.82, p=.024** | b=.59, p=.500 | b=.96, p=.204 | b=-.14, p=.441 | b=.78, p=.195 | b=.69, p=.124 |
| Outcome on | | | | | | |
| Sex | **b=.90, p=.019** | **b=.90, p=.004** | **b=1.47, p=.003** | **b=.85, p=.008** | **b=.85, p=.009** | **b=.94, p=.003** |
| Risk | **b=2.48, p<.001** | **b=2.48, p<.001** | **b=2.52, p<.001** | **b=2.43, p<.001** | **b=2.52, p<.001** | **b=2.52, p<.001** |
| Intercept | b=-.04, p=.262 | b=-.08, p=.195 | b=.11, p=.106 | b=-.04, p=.911 | b=.09, p=.162 | b=.23, p=.100 |
| Slope | b=-.05, p=.595 | b=.04, p=.747 | b=-.19, p=.251 | b=.21, p=.531 | b=.23, p=.354 | b=.15, p=.406 |

Supplementary Materials Table 5: Test of risk and risk*site interaction term in predicting intercept and slope of absolute EEG Power.

|  | **Delta Intercept** | **Theta Intercept** | **Low Alpha Intercept** | **High Alpha Intercept** | **Beta Intercept** | **Gamma Intercept** |
| --- | --- | --- | --- | --- | --- | --- |
| **Risk** | b=-8.31, p=.006 | b=-11.27, p=.002 | b=-9.78, p=.003 | b=-10.23, p<.001 | b=-9.62, p<.001 | b=-11.14, p<.001 |
| **Risk*Site** | χ^2^(2)=0.92, p=.631 | χ^2^(2)=1.83, p=.401 | χ^2^(2)=1.82, p=.402 | χ^2^(2)=1.82, p=.402 | χ^2^(2)=0.15, p=.927 | χ^2^(2)=2.08, p=.354 |
|  | **Delta Slope** | **Theta Slope** | **Low Alpha Slope** | **High Alpha Slope** | **Beta Slope** | **Gamma Slope** |
| **Risk** | b=2.86, p=.050 | b=3.74, p=.036 | b=3.51, p=.062 | b=3.81, p=.010 | b=3.77, p=.008 | b=3.55, p=.017 |
| **Risk*Site** | χ^2^(2)=1.78 p=.411 | χ^2^(2)=1.09, p=.580 | χ^2^(2)=0.58, p=.749 | χ^2^(2)=1.46, p=.481 | χ^2^(2)=1.81, p=.405 | χ^2^(2)=2.26, p=.323 |

Supplementary Materials Figure 1: estimated trajectories of absolute power for each participant based on structural equation model.

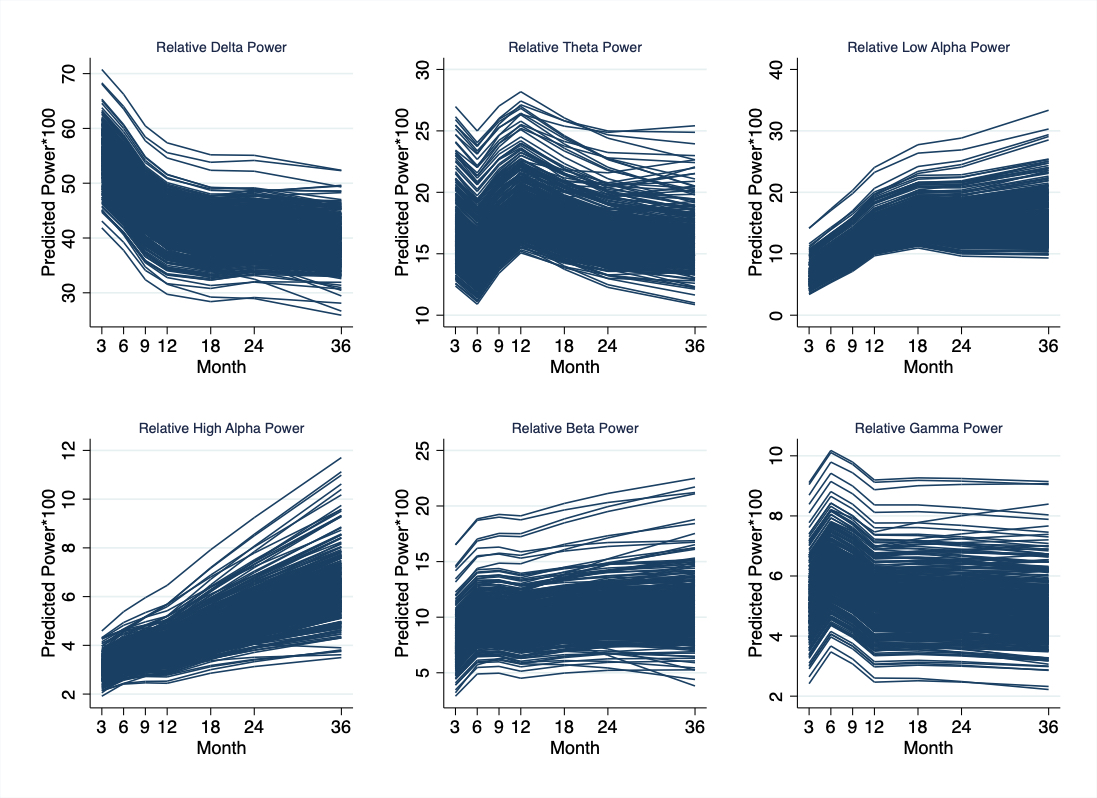
Supplementary Materials Figure 2: estimated trajectories of relative power for each participant based on structural equation model.
